# SGLT2i use and fracture risk compared with DPP4i in patients with T2DM

**DOI:** 10.1111/jdi.70433

**Published:** 2026-09-16

**Authors:** Sung‐Yen Lin, Shih‐Hao Huang, Ching‐Han Chiang, Cheng‐Jung Ho, Chung‐Hwan Chen, Li‐Nien Chien

**Affiliations:** ^1^ School of Post‐Baccalaureate Medicine, College of Medicine Kaohsiung Medical University Kaohsiung Taiwan; ^2^ Department of Orthopedics, Kaohsiung Medical University Gangshan Hospital Kaohsiung Medical University Kaohsiung Taiwan; ^3^ Division of Adult Reconstruction Surgery, Department of Orthopedics, Kaohsiung Medical University Hospital Kaohsiung Medical University Kaohsiung Taiwan; ^4^ Orthopaedic Research Center Kaohsiung Medical University Kaohsiung Taiwan; ^5^ Regenerative Medicine and Cell Therapy Research Center Kaohsiung Medical University Kaohsiung Taiwan; ^6^ Institute of Health and Welfare Policy, College of Medicine National Yang Ming Chiao Tung University Taipei Taiwan; ^7^ School of Medicine, College of Medicine Kaohsiung Medical University Kaohsiung Taiwan; ^8^ Department of Orthopedics, College of Medicine Kaohsiung Medical University Kaohsiung Taiwan; ^9^ Graduate Institute of Materials Engineering, College of Engineering National Pingtung University of Science and Technology Pingtung Taiwan; ^10^ Department of Healthcare Administration and Medical Informatics Kaohsiung Medical University Kaohsiung Taiwan; ^11^ Institute of Medical Science and Technology National Sun Yat‐Sen University Kaohsiung Taiwan; ^12^ Graduate Institute of Animal Vaccine Technology, College of Veterinary Medicine National Pingtung University of Science and Technology Pingtung Taiwan; ^13^ Program in Biomedical Engineering, College of Medicine Kaohsiung Medical University Kaohsiung Taiwan

**Keywords:** fracture risk, SGLT2i, T2DM

## Abstract

**Introduction:**

The association between sodium‐glucose cotransporter‐2 inhibitor (SGLT2i) use and fracture risk remains inconclusive. Most previous studies have focused on treatment‐naïve patients, despite many patients in routine clinical practice initiating SGLT2i therapy after prior dipeptidyl peptidase‐4 inhibitor (DPP4i) use. This study aimed to evaluate the risk of fractures associated with SGLT2i compared with dipeptidyl peptidase‐4 inhibitors (DPP4i) in Taiwan.

**Materials and Methods:**

This nationwide cohort study used Taiwan's National Health Insurance Research Database. Patients with T2DM initiating SGLT2i or DPP4i therapy between 2016 and 2019 were identified. Follow‐up began one year after the index date to ensure adequate treatment exposure before outcome assessment. A prevalent new‐user design and 1:2 propensity score matching (PSM) were applied to balance baseline characteristics. Fractures were ascertained from medical claims, and competing risk regression was used to estimate fracture risk, accounting for death as a competing event.

**Results:**

After PSM, 106,992 SGLT2i users and 213,984 DPP4i users were included with comparable baseline profiles. Over a mean follow‐up of 3.5 years, the overall risk of any fracture did not differ between groups. Although SGLT2i use was associated with a significantly lower risk of hip fracture compared with DPP4i use (subdistribution hazard ratio = 0.89, 95% CI: 0.81–0.98), this association was no longer statistically significant after adjustment for multiple comparisons. Subgroup analyses yielded generally consistent findings.

**Conclusions:**

In this cohort, SGLT2i use was not associated with increased overall or major osteoporotic fracture risk vs DPP4i. The prevalent new‐user design captures prior treatment histories and reflects real‐world practice.

## INTRODUCTION

Sodium‐glucose cotransporter 2 inhibitors (SGLT2i) have marked a significant advancement in the treatment of type 2 diabetes mellitus (T2DM) since their approval by the US Food and Drug Administration (FDA) in 2013. Initially developed to lower glucose through urinary excretion, SGLT2i have demonstrated substantial benefits beyond glycemic control. Landmark clinical trials, including EMPA‐REG OUTCOME (Empagliflozin, Cardiovascular Outcomes, and Mortality in Type 2 Diabetes)^1^ and CREDENCE (Canagliflozin and Renal Events in Diabetes with Established Nephropathy Clinical Evaluation)^2^, have shown that SGLT2i significantly reduce major adverse cardiovascular events and slow the progression of chronic kidney disease (CKD) in patients with type 2 diabetes mellitus. These findings have established SGLT2i as a preferred therapeutic option, particularly for patients at elevated cardiovascular or renal risk.

SGLT2i has been rapidly adopted worldwide. In the United States, annual SGLT2i prescriptions doubled between 2015 and 2020. In the United Kingdom, SGLT2i accounted for 22% of new antidiabetic prescriptions by 2017, reflecting growing recognition of their clinical benefits^3^. However, concerns about bone health have emerged^4^. The CANVAS program^5^, which evaluated canagliflozin, reported an increased risk of bone fractures, whereas the EMPA‐REG OUTCOME trial found no elevated fracture risk associated with empagliflozin compared to placebo. These contrasting results underscore the need to clarify fracture risk within the SGLT2i class.

Although several real‐world studies using claims data or electronic health records from the United States, the United Kingdom, and Korea have not demonstrated a significant association between SGLT2i use and fracture risk, these studies primarily employed a new‐user design that may not reflect real‐world patterns in Taiwan, where many patients initiate SGLT2i therapy after extended dipeptidyl peptidase‐4 inhibitor (DPP4i) use. Evidence suggests that DPP4i use may be associated with modestly higher bone mineral density and a lower risk of osteoporosis, raising the possibility that prior DPP4i exposure may influence baseline skeletal health at the time of SGLT2i initiation. Consequently, fracture risk after transitioning from DPP4i to SGLT2i may differ from that observed in treatment‐naïve patients, highlighting the need to evaluate this common treatment sequence in routine clinical practice. Moreover, the relatively short follow‐up periods in those studies may have limited the ability to capture longer‐term fracture outcomes. Therefore, we applied a prevalent new‐user design to evaluate the association between SGLT2i use and fracture risk in routine clinical practice.

## METHODS

### Ethics statement

This study was approved by the Institutional Review Board of National Yang Ming Chiao Tung University (IRB No. NYCU113050AE). Data confidentiality was maintained in accordance with Health and Welfare Data Science Center regulations. As all data were de‐identified and encrypted, the requirement for informed consent was waived. All procedures complied with IRB and relevant regulatory standards.

### Data sources

We used the National Health Insurance Research Database (NHIRD), a nationwide claims‐based database maintained by the National Health Insurance Administration (NHIA) of Taiwan. The NHIRD derives from Taiwan's mandatory single‐payer insurance program, covering over 99% of the population since 1995. The database includes claims, diagnoses, procedures, prescriptions, and healthcare utilization across outpatient, inpatient, and emergency settings. For this study, data from 2011 to 2022 included diagnoses coded using the International Classification of Diseases, Ninth and Tenth Revision, Clinical Modification (ICD‐9‐CM and ICD‐10‐CM), medication records classified under the Anatomical Therapeutic Chemical (ATC) system, demographic data, reimbursement information, and healthcare provider identifiers. Mortality was ascertained through linkage to the National Death Registry.

### Study design

This study adopted a prevalent new‐user cohort design to evaluate SGLT2i against established therapies. This design captures patients transitioning from older glucose‐lowering therapies to newer agents, thereby better reflecting real‐world clinical practice and enhancing the generalizability of safety assessments^6^. DPP4i were selected as the comparator for several reasons. First, before the introduction of SGLT2i, DPP4i were commonly prescribed second‐line oral antidiabetic drugs (OADs) in Taiwan. Many patients initiating SGLT2i therapy had prior exposure to DPP4i or other OADs. Second, DPP4i have a well‐established safety profile, making them a clinically appropriate comparator. Third, previous comparative studies have shown that patients initiating SGLT2i and DPP4i have comparable baseline HbA1c levels and achieve similar glycemic control after treatment, supporting their use as an active comparator^7^. Additionally, until 2019, Taiwan's National Health Insurance (NHI) policy prohibited the concurrent use of SGLT2i and DPP4i, further justifying the use of DPP4i as a reference group in evaluating the switch to SGLT2i therapy.

### Study cohort

**Figure 1:**
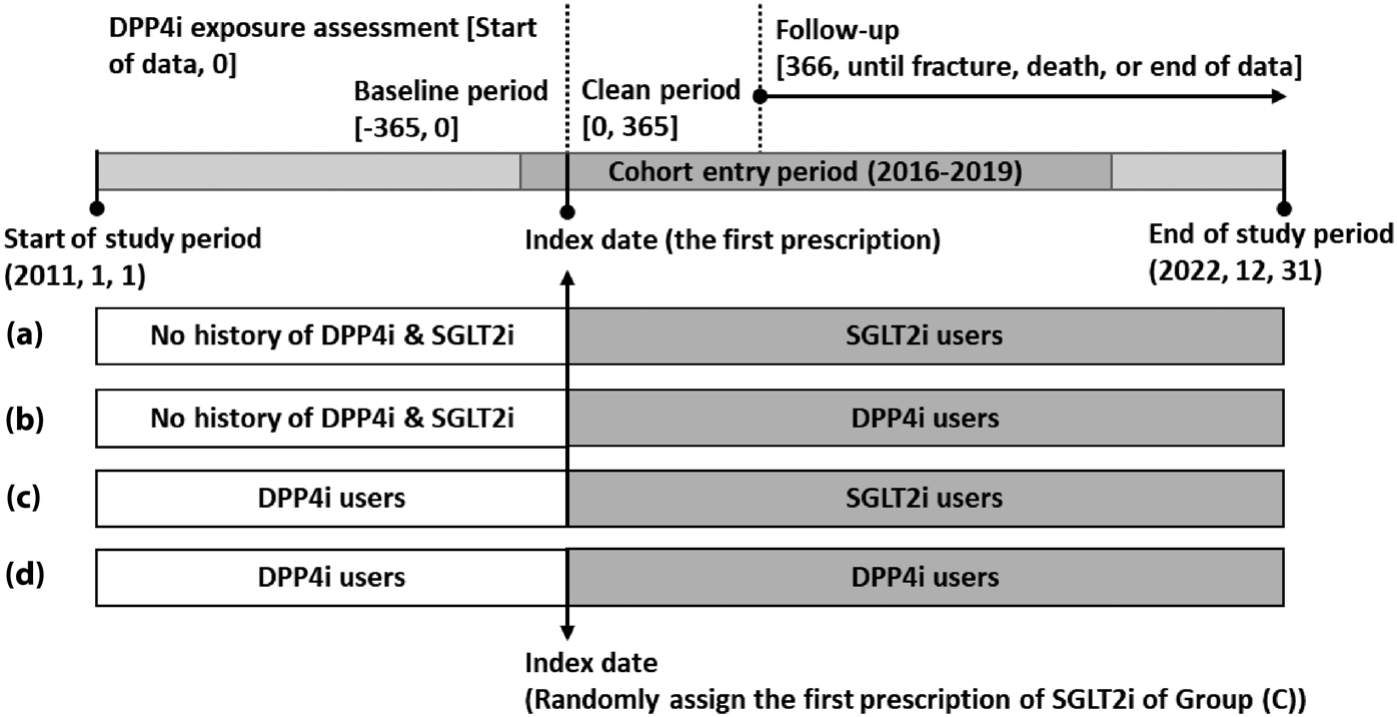
Time schema and index date for the four study groups under the prevalent new‐user design. DPP4i, dipeptidyl peptidase‐4 inhibitor; SGLT2i, sodium‐glucose cotransporter‐2 inhibitor.

We identified patients with T2DM who initiated SGLT2i (ATC code: A10BK) or DPP4i (ATC code: A10BH) between 2016 and 2019. Figure 1 shows the data schema and index date assignment. During the study period, the available SGLT2i included dapagliflozin and empagliflozin, which became reimbursable in May 2016, and canagliflozin, which became reimbursable starting in January 2019.

**Figure 2:**
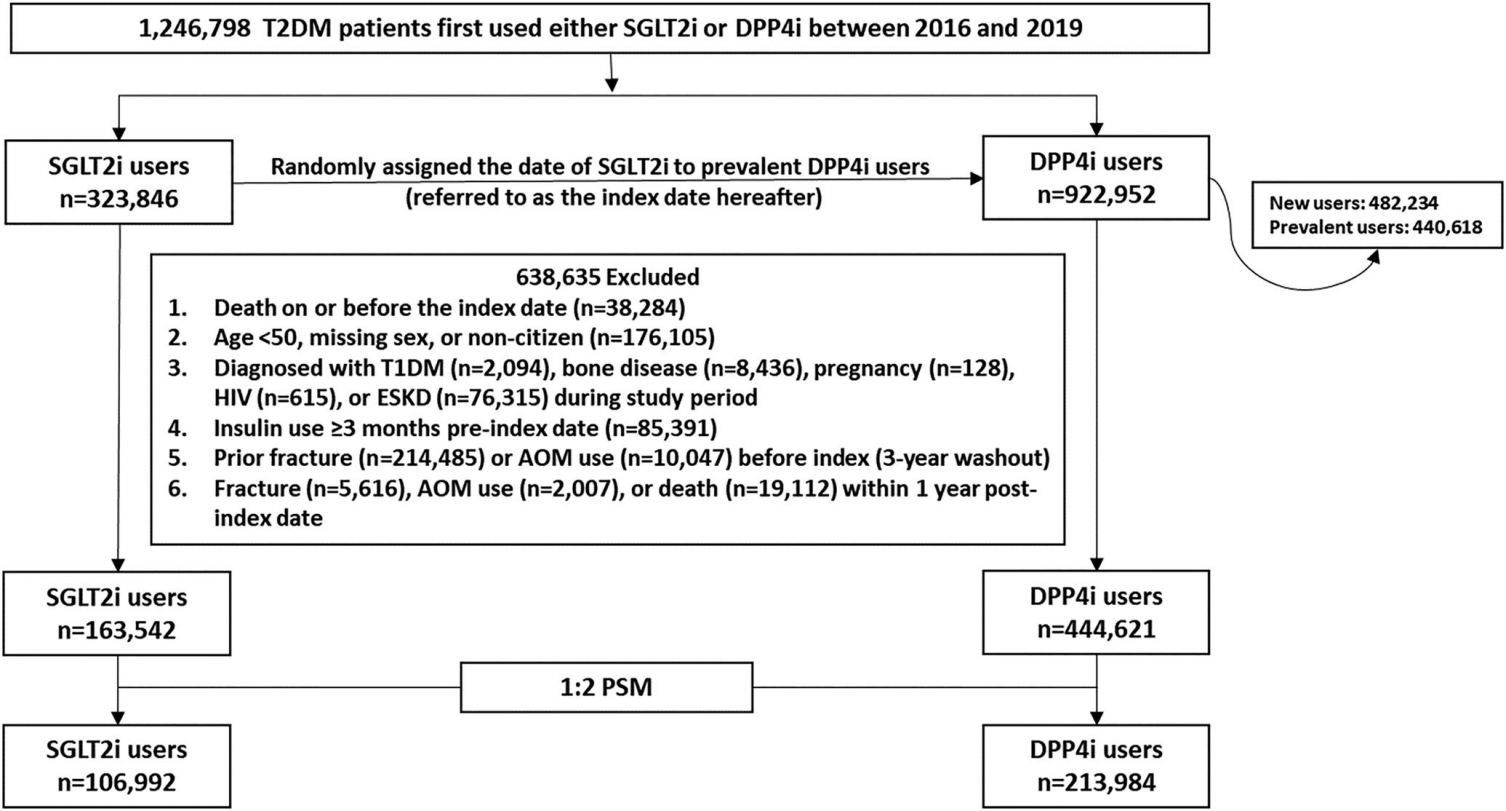
Patient selection flow. AOM, anti‐osteoporotic medication; DPP4i, dipeptidyl peptidase‐4 inhibitor; ESKD, end‐stage kidney disease; HIV, human immunodeficiency virus; PSM, propensity score matching; SGLT2i, sodium‐glucose cotransporter‐2 inhibitor; T1DM, type 1 diabetes mellitus; T2DM, type 2 diabetes mellitus.

The study cohort was categorized into four groups: (A) incident new users of SGLT2i, (B) incident new users of DPP4i, (C) prevalent new users of SGLT2i, and (D) prevalent users of DPP4i, as depicted in Figure 1. The index date was defined as the date of the first prescription of SGLT2i or DPP4i for Groups A‐C. For prevalent DPP4i users (Group D), we adopted the time‐based approach proposed by Suissa *et al*.^6^ Prevalent DPP4i users were first matched to prevalent new SGLT2i users using the same index year and duration of prior DPP4i exposure. The corresponding index date of the matched prevalent new SGLT2i user was then assigned as the pseudo‐index date for the prevalent DPP4i user to align the calendar time and treatment history before baseline covariate assessment. Figure 2 illustrates the patient selection process. We first excluded individuals who died on or before the index date, due to the random assignment of index dates in Group D. Additional exclusions were applied to patients younger than 50 years, those with missing sex information, or non‐citizens of Taiwan. To eliminate individuals with known risk factors for fracture, we excluded patients with type 1 diabetes mellitus (T1DM), metastatic bone disease, pregnancy, HIV infection, or end‐stage kidney disease (ESKD). Patients with continuous insulin use for at least 3 months before the index date were also excluded. A 3‐year washout period was implemented to exclude those with a history of fractures or anti‐osteoporotic medication (AOM) use. In Taiwan, reimbursement for AOMs is limited to patients with confirmed osteoporotic fractures; therefore, prior AOM use strongly indicates a history of fragility fractures, justifying their exclusion from the study cohort. To ensure adequate ascertainment of treatment exposure before outcome assessment, follow‐up began 1 year after the index date. This prespecified one‐year clean period allowed sufficient time for sustained treatment exposure before fracture risk evaluation and reduced the likelihood that fractures occurring shortly after treatment initiation reflected underlying skeletal fragility or events occurring before adequate medication exposure had been established. Therefore, patients who experienced a fracture, initiated anti‐osteoporotic medication, or died during this 1‐year period were excluded from the study cohort.

### Fracture outcomes

This study focused on identifying new‐onset fractures, with particular attention to osteoporotic fractures due to their potential association with the biochemical effects of SGLT2i. Fractures were ascertained from inpatient and outpatient claims using specific ICD‐10‐CM diagnosis codes and procedure codes related to fracture management and surgical intervention, consistent with methods applied in prior studies^8^, ^9^ (Table S1). Fractures associated with high‐energy trauma (e.g., traffic accidents or major injuries) were excluded based on accompanying trauma‐related diagnosis codes, ensuring that only fragility fractures were analyzed.

Two algorithms were used for fracture identification. The first relied on hospitalization records, with or without accompanying surgical procedures. This approach has been validated in previous studies using Taiwan's healthcare data for identifying incident hip fractures, demonstrating a positive predictive value (PPV) exceeding 90%^10^. While the same algorithm was applied to other fracture types, formal validation for those sites is lacking. The second algorithm was based solely on outpatient claims and required at least three fracture‐related diagnoses within 12 months. This method, adapted and modified from a previous study to reflect clinical practices and coding conventions in Taiwan, has demonstrated moderate PPV.

### Covariates

To balance baseline characteristics between the SGLT2i and DPP4i groups, we included covariates related to demographics, diabetes severity, comorbidities (identified using ICD‐9‐CM or ICD‐10‐CM codes; see Table S2), and concurrent medication use (classified by ATC codes; see Table S3). These variables were selected based on their established associations with fracture risk and the choice of OADs. Diabetic duration was defined as the interval between the first recorded T2DM diagnosis and the index date. Because the true onset date of diabetes could precede the first record in the claims database, the exact duration of diabetes prior to cohort entry was unknown. Therefore, the duration estimated from the first available diagnosis record represents an observable proxy rather than the true clinical duration. Diabetes severity was assessed using the adapted Diabetes Complications Severity Index (aDCSI), a claims‐based tool widely used to quantify the burden of diabetes‐related complications. We also considered the use of second‐line OADs within 3 months before follow‐up, as some agents, such as SU and TZD, have been linked to increased fracture risk.

Comorbid conditions associated with fracture risk were included, such as osteoporosis, hyperlipidemia, hypertension, chronic liver disease, diabetic retinopathy, prior myocardial infarction, stroke, heart failure, arrhythmia, peripheral artery occlusion, and polyneuropathy. In addition, we adjusted for the use of medications that may affect bone metabolism, muscle coordination, or fall risk, including antihypertensives (ACE inhibitors/ARBs, beta‐blockers, calcium channel blockers), antiplatelet agents, and corticosteroids.

### Statistical analysis

We conducted survival analyses to evaluate the association between SGLT2i use and fracture risk. The follow‐up period began 1 year after the index date and continued until the first occurrence of fracture, death, or December 31, 2022, whichever came first. An intention‐to‐treat (ITT) approach was used in the primary analysis. To account for the competing risk of death, we applied a Fine‐Gray subdistribution hazard model to estimate subdistribution hazard ratios (SHRs) in the PSM cohort without additional covariate adjustment, as baseline characteristics were well balanced after propensity score matching.

To improve comparability, 1:2 propensity score matching (PSM) was performed using a greedy matching algorithm without replacement. The propensity score model included all baseline covariates described in the ‘Covariates’ section. A caliper width of 0.01 on the logit of the propensity score was applied, with exact matching on index year and duration of prior DPP4i exposure to ensure temporal alignment and comparable treatment history between matched patients. Baseline characteristics between SGLT2i and DPP4i users were compared using standardized mean differences (SMD), with an SMD >0.1 indicating a meaningful imbalance. We also conducted subgroup analyses stratified by age, sex, new or prevalent users, and severity of T2DM. Two sensitivity analyses were performed to test the robustness of our findings. First, inverse probability of treatment weighting (IPTW) based on propensity scores was performed as a sensitivity analysis. Covariate balance after weighting was reassessed. No trimming or truncation of extreme weights was applied. Second, an as‐treated analysis was conducted to account for medication discontinuation, switching, or add‐on therapy during follow‐up. Medication discontinuation was defined as a gap of more than 60 days between two consecutive prescriptions (grace period). Treatment switching was defined as initiation of the alternative study drug class, whereas add‐on therapy was defined as initiation of the alternative study drug class while continuing the initial study drug. In the as‐treated analysis, follow‐up was additionally censored at treatment discontinuation, switching, or add‐on therapy, in addition to the censoring criteria used in the primary analysis. All analyses were conducted using SAS version 9.4 and SAS/STAT® version 15.2 (SAS Institute, Cary, NC, USA). A two‐sided P value <0.05 was considered statistically significant.

## RESULTS

### Baseline characteristics

Following the application of exclusion criteria, 163,542 SGLT2i users and 444,621 DPP4i users were identified between 2016 and 2019. Before matching, significant differences in baseline characteristics were observed between the two groups (Table 1). Compared to DPP4i users, SGLT2i users were younger (mean age 62.3 vs. 66.3 years), included a lower proportion of women (42 vs. 45%), and had a longer history of diabetes (6.1 vs. 5.6 years). Comorbidities also differed, with a higher prevalence of ischemic heart disease (19.6 vs. 14.4%) and a lower prevalence of dementia (1.0% vs. 2.7%) among SGLT2i users.

**Table 1:** Baseline characteristics of DPP4i and SGLT2i users before and after PSM

	Before PSM			After PSM		
	DPP4i	SGLT2i		DPP4i	SGLT2i	
	(%)	(%)	SMD^‡^	(%)	(%)	SMD^‡^
Sample Size	444,621	163,542		213,984	106,992	
Female	(44.7)	(41.8)	0.059	(44.6)	(44.6)	<0.001
Age, y						
mean, SD	(66.3, 9.8)	(62.7, 8.2)	0.402	(64.7, 8.3)	(64.7, 8.3)	0.002
50–64	(47.0)	(61.8)	0.302	(51.3)	(51.4)	0.001
65–74	(31.6)	(28.9)	0.058	(35.4)	(35.4)	0.001
75+	(21.4)	(9.2)	0.343	(13.3)	(13.3)	0.001
Year of cohort entry						
2016	(21.6)	(15.6)	0.156	(14.6)	(14.6)	<0.001
2017	(25.0)	(23.9)	0.026	(24.0)	(24.0)	<0.001
2018	(25.6)	(26.5)	0.022	(27.8)	(27.8)	<0.001
2019	(27.8)	(34.0)	0.135	(33.5)	(33.5)	<0.001
DM duration, years (mean, SD)	(5.6, 3.0)	(6.1, 2.9)	0.177	(6.0, 2.9)	(6.0, 2.9)	0.014
DM‐related comorbidities						
Diabetic nephropathy	(22.6)	(23.0)	0.011	(22.2)	(23.3)	0.028
Diabetic neuropathy	(9.3)	(9.6)	0.010	(9.2)	(9.6)	0.014
Diabetic retinopathy	(5.7)	(6.3)	0.025	(6.1)	(6.3)	0.010
Hyperglycemia	(0.8)	(0.5)	0.039	(0.5)	(0.5)	0.006
Hypoglycemia	(0.7)	(0.3)	0.051	(0.3)	(0.4)	0.012
aDCSI (mean, SD)	(1.8, 2.3)	(1.9, 2.4)	0.043	(1.8, 2.3)	(1.8, 2.3)	0.030
Other comorbidity conditions						
Hypertension	(61.9)	(64.0)	0.043	(61.4)	(64.1)	0.056
Hyperlipidemia	(26.9)	(30.0)	0.068	(27.1)	(26.4)	0.016
Ischemic heart disease	(14.4)	(19.6)	0.140	(13.5)	(15.7)	0.063
Cardiac arrhythmia	(0.8)	(0.6)	0.033	(0.5)	(0.6)	0.014
HF	(4.5)	(5.2)	0.035	(3.7)	(4.6)	0.046
PVD	(3.5)	(3.3)	0.011	(3.4)	(3.6)	0.013
Ischemic stroke	(6.6)	(4.3)	0.099	(5.1)	(5.6)	0.026
CKD	(7.5)	(5.6)	0.076	(6.1)	(6.9)	0.032
CLD	(7.7)	(6.8)	0.036	(6.8)	(7.7)	0.035
Cancer	(6.6)	(4.7)	0.081	(5.7)	(6.0)	0.014
Dementia	(2.7)	(1.0)	0.129	(0.9)	(1.4)	0.045
Hypovolemia	(0.3)	(0.2)	0.027	(0.2)	(0.2)	0.007
Osteoporosis	(0.9)	(0.7)	0.028	(0.8)	(0.8)	0.006
Modified CCI (mean, SD)	(2.3, 1.7)	(2.2, 1.5)	0.038	(2.2, 1.6)	(2.3, 1.6)	0.038
Anti‐diabetes drugs						
Metformin	(70.5)	(85.4)	0.364	(79.9)	(79.1)	0.019
SU	(47.9)	(62.8)	0.302	(53.4)	(53.6)	0.005
GLP1‐RA	(0.3)	(0.7)	0.047	(0.3)	(0.3)	0.004
Meglitinides	(3.8)	(3.4)	0.024	(3.5)	(3.8)	0.016
TZD	(7.9)	(13.4)	0.181	(7.9)	(8.2)	0.011
α‐glucosidase inhibitors	(9.4)	(13.3)	0.122	(9.6)	(10.2)	0.021
Insulin	(3.8)	(6.9)	0.141	(3.5)	(3.9)	0.020
Other drugs						
ACEI	(3.3)	(3.4)	0.004	(3.3)	(3.3)	<0.001
ARB	(24.2)	(27.5)	0.076	(24.7)	(25.8)	0.025
α‐blockers	(3.4)	(3.2)	0.010	(3.1)	(3.6)	0.023
β‐blockers	(24.6)	(29.5)	0.109	(24.5)	(26.5)	0.046
CCB	(28.5)	(27.7)	0.018	(27.6)	(30.1)	0.055
Loop diuretics	(3.3)	(2.8)	0.031	(2.4)	(3.1)	0.044
Thiazide	(1.5)	(1.4)	0.008	(1.3)	(1.6)	0.019
Potassium‐sparing agents	(1.9)	(2.3)	0.026	(1.7)	(2.0)	0.023
Antiplatelets	(28.5)	(31.8)	0.071	(27.5)	(30.6)	0.069
Statins	(43.2)	(54.5)	0.227	(46.7)	(47.2)	0.009
NSAIDs	(82.9)	(85.6)	0.075	(83.2)	(85.2)	0.053
Healthcare utilization						
Any hospitalization [−90, 0]	(10.0)	(6.0)	0.147	(6.8)	(7.2)	0.016
Any hospitalization [−365, −91]	(10.0)	(9.4)	0.020	(8.9)	(9.6)	0.023
Outpatient visits [−365, 0], (mean, SD)	(20.1, 14.7)	(19.6, 13.6)	0.038	(19.6, 14.0)	(20.5, 14.4)	0.058
Prior DPP4i exposure duration, y (mean, SD)^†^	(1.1, 1.3)	(1.3, 1.2)	0.222	(1.2, 1.3)	(1.2, 1.2)	0.026
Follow‐up period, years (mean, SD)	(3.7, 1.3)	(3.6, 1.1)	0.058	(3.5, 1.2)	(3.5, 1.1)	0.005

aDCSI, adapted Diabetes Complications Severity Index; ACEI, angiotensin‐converting enzyme inhibitor; ARB, angiotensin II receptor blocker; CCB, calcium channel blocker; CCI, Charlson comorbidity index; CKD, chronic kidney disease; CLD, chronic lung disease; DM, diabetes mellitus; DPP4i, dipeptidyl peptidase‐4 inhibitor; GLP1‐RA, glucagon‐like peptide‐1 receptor agonist; HF, heart failure; NSAIDs, nonsteroidal anti‐inflammatory drugs; PSM, propensity score matching; PVD, peripheral vascular disease; SD, standard deviation; SGLT2i, sodium‐glucose cotransporter‐2 inhibitor; SMD, standardized mean difference; SU, sulfonylurea; TZD, thiazolidinedione.

^‡^ SMD <0.1 denotes balance in the covariate between the two groups.

^†^ Prior DPP4i exposure refers to DPP4i exposure before the applicable index date or pseudo‐index date.

In terms of medication use, SGLT2i users were more likely to have used metformin, SU, TZD, α‐glucosidase inhibitors, insulin, β‐blockers, and statins. They also had fewer hospitalizations before the index date and a longer duration of prior DPP4i exposure (1.3 vs. 1.1 years).

After applying 1:2 propensity score matching, the final cohort consisted of 106,992 SGLT2i users and 213,984 DPP4i users. Baseline characteristics were well balanced between groups, with SMD for all covariates reduced to below 0.1, indicating successful matching (Table 1).

### Incidence and risk of fracture

**Figure 3:**
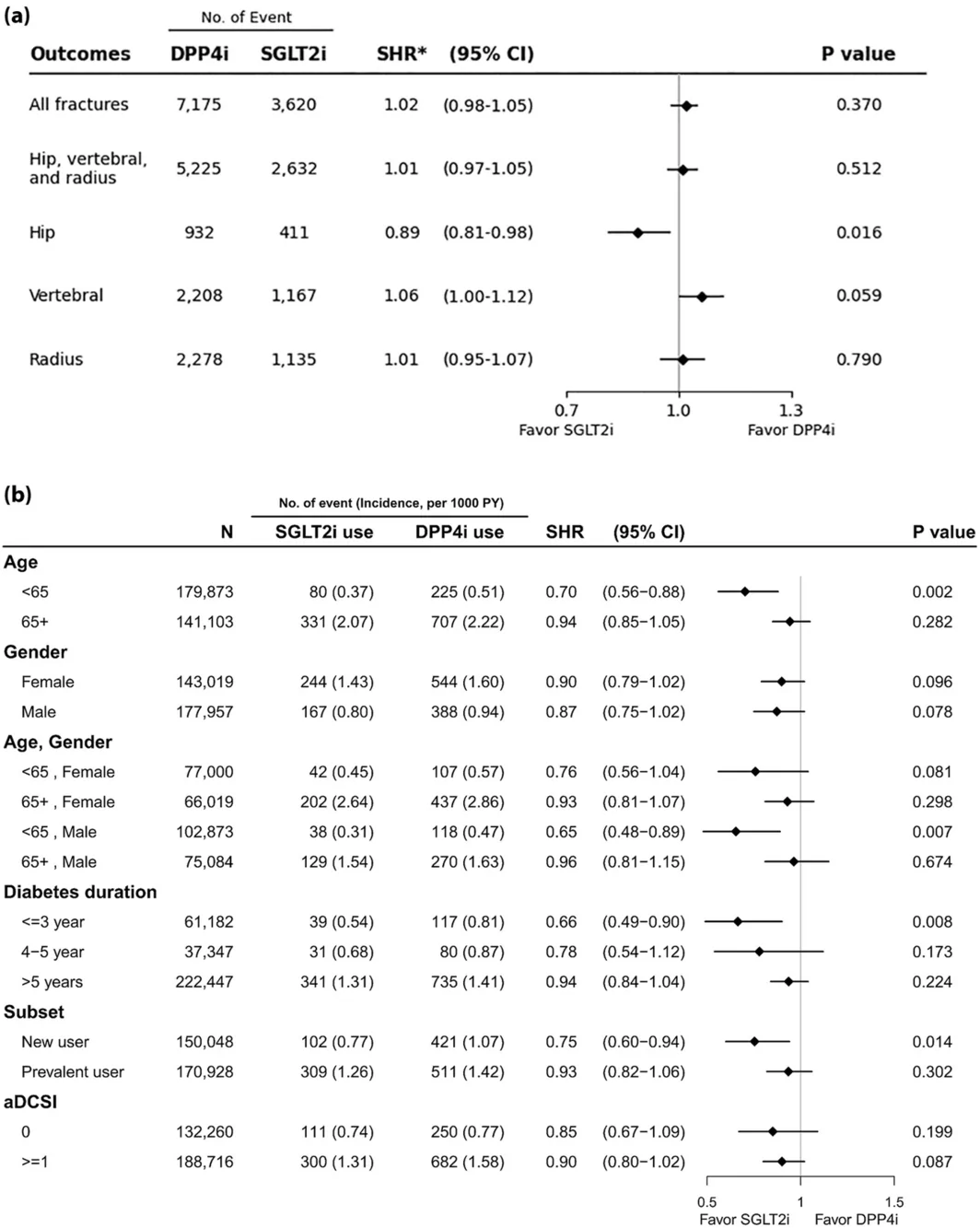
(a) Subdistribution hazard ratios for fracture outcomes. *SHR was estimated based on conditional competing risk model analysis. CI, confidence interval; DPP4i, dipeptidyl peptidase‐4 inhibitor; SHR, subdistribution hazard ratio; SGLT2i, sodium‐glucose cotransporter‐2 inhibitor. (b) Subgroup analysis of hip fracture risk in T2DM patients treated with SGLT2i vs DPP4i. aDCSI, adapted Diabetes Complications Severity Index; CI, confidence interval; DPP4i, dipeptidyl peptidase‐4 inhibitor; SGLT2i, sodium‐glucose cotransporter‐2 inhibitor; SHR, subdistribution hazard ratio; T2DM, type 2 diabetes mellitus.

Over a mean follow‐up period of 3.5 years (SD = 1.1), SGLT2i users did not exhibit a higher incidence of overall fractures compared to DPP4i users. The incidence rate of all fractures was 9.72 per 1,000 person‐years (PY) (95% confidence interval [CI], 9.41–10.00) in the SGLT2i group and 9.65 per 1,000 PY (95% CI, 9.43–9.88) in the DPP4i group (Table S4). Using a competing risk model, the SHR for all fractures was 1.02 (95% CI, 0.98–1.05), indicating no significant difference between groups. Similarly, no difference was observed in the risk of osteoporotic fractures—including hip, vertebral, and radial fractures. Notably, however, SGLT2i users had a significantly lower risk of hip fracture than DPP4i users, with an SHR of 0.89 (95% CI, 0.81–0.98) (Figure 3a).

### Subgroup analysis

Figure 3b presents a forest plot displaying the number of events, sample sizes, and SHR (SHR) for hip fracture across various subgroups. A significantly lower risk of hip fracture was observed among T2DM patients aged <65 years (SHR = 0.70; 95% CI, 0.56–0.88), males aged <65 years (SHR = 0.65; 95% CI, 0.48–0.89), those with a DM duration of ≤3 years (SHR = 0.66; 95% CI, 0.49–0.90), and new SGLT2i users (SHR = 0.75; 95% CI, 0.60–0.94). However, formal tests for interaction were not statistically significant, suggesting no evidence that the treatment association differed across these subgroup characteristics.

### Sensitivity analysis

After applying IPTW, the weighted sample sizes for the SGLT2i and DPP4i groups were 598,480 and 609,710, respectively, and all baseline covariates were well balanced (all SMDs <0.1). In the as‐treated analysis, approximately 30% of DPP4i users and 50% of SGLT2i users either switched to or added the alternative therapy during follow‐up. Importantly, both the IPTW and as‐treated analyses produced SHRs consistent with the primary findings (Tables S5 and S6), reinforcing the robustness of the results.

## DISCUSSION

To the best of our knowledge, this is the first real‐world, nationwide cohort study using a prevalent user design to compare fracture risk between SGLT2i and DPP4i users in patients with T2DM. Leveraging comprehensive data from Taiwan's NHIRD, we assessed both overall and site‐specific fracture outcomes. After PSM, our analysis demonstrated that SGLT2i use was not associated with an increased risk of overall or major osteoporotic fractures compared to DPP4i use. In the overall cohort, SGLT2i use was associated with a lower risk of hip fracture (SHR 0.89; 95% CI, 0.81–0.98), whereas no significant differences were observed for vertebral or radial fractures. Subgroup analyses showed lower SHRs among incident new users, younger men (<65 years), and patients with a diabetes duration ≤3 years. However, formal tests for interaction were not statistically significant, indicating no evidence of effect modification across these subgroup characteristics.

SGLT2i provide important cardiovascular, renal, and metabolic benefits, but their skeletal safety has been debated since the CANVAS trial, which reported a higher fracture incidence with canagliflozin^5^. Proposed mechanisms include falls related to volume depletion, weight loss–associated bone loss, increased bone turnover, and disturbed mineral homeostasis^11^, ^12^. Yet, subsequent RCTs and meta‐analyses, including those in high‐risk groups, have not consistently confirmed this signal^13^, ^14^, ^15^, ^16^. Notably, two large population‐based studies using DPP4i as comparators also showed no excess fracture risk, although DPP4i may exert incretin‐mediated skeletal protection^17^, ^18^. Such effects could potentially obscure differences in fracture risk between treatment groups. In addition, evidence suggests that DPP4i use may be associated with modestly higher bone mineral density and a lower risk of osteoporosis, raising the possibility that prior DPP4i exposure may influence baseline skeletal health^19^. Moreover, while most prior studies involved treatment‐naïve patients, real‐world practice often involves switching from DPP4i to SGLT2i, meaning that patients initiating SGLT2i may differ from treatment‐naïve patients with respect to baseline skeletal health and cumulative glycemic exposure. This clinically relevant treatment sequence has been insufficiently evaluated in previous studies. To address this evidence gap, we applied a prevalent new‐user design with additional matching on the duration of prior DPP4i exposure, thereby better reflecting routine clinical practice and providing evidence regarding fracture risk after transitioning from DPP4i to SGLT2i therapy.

Although bone mineral density (BMD) is widely used to assess fracture risk, it does not fully capture bone quality, particularly microarchitectural integrity. This is evident in T2DM, where fracture risk is increased despite preserved BMD (‘diabetic paradox of bone fragility’^18^). This paradox is largely attributed to cortical microarchitectural deterioration independent of BMD. Imaging studies suggest that T2DM predominantly affects cortical bone, whereas trabecular deficits are more prominent in T1DM^20^, ^21^. Given the key role of cortical bone in hip strength, the increased cortical porosity observed in T2DM may disproportionately weaken the hip compared to trabecular‐dominant sites such as the spine^22^. Consistent epidemiological evidence shows higher hip fracture risk in T2DM without corresponding increases at vertebral or distal forearm sites^23^. Our finding of a lower risk of hip fracture, in the absence of significant differences in vertebral or radial fractures, may reflect preferential cortical deterioration in T2DM and the distinct biomechanical roles of cortical and trabecular bone.

Although lower SHRs were observed among younger patients and those with shorter diabetes duration, formal interaction tests did not provide evidence of effect modification. Nevertheless, the biological mechanisms discussed above may plausibly explain the observed subgroup patterns. These findings should be considered hypothesis‐generating and require confirmation in future studies. Chronic hyperglycemia is strongly implicated in impaired bone quality and microarchitectural deterioration, primarily through the accumulation of advanced glycation end products (AGEs). AGE‐mediated collagen crosslinking disrupts mineralization and impairs bone mechanical properties^24^. AGEs also impair osteoblast function and promote oxidative stress^24^, ^25^. Because AGE accumulation reflects cumulative hyperglycemic exposure, early glycemic control may preserve bone integrity. SGLT2i have demonstrated greater glucose‐lowering efficacy than DPP4i when combined with metformin, suggesting a more favorable impact on long‐term glycemic exposure^26^. Longitudinal imaging data further suggest that cortical deterioration may be most pronounced during early T2DM^27^. Together, these findings suggest that early SGLT2i initiation may mitigate cortical deterioration during a potentially vulnerable phase of diabetic bone remodeling.

While clinical evidence for the skeletal benefits of SGLT2i remains limited, animal studies provide growing evidence of their potential to preserve bone quality in diabetes. In a T1DM mouse model, canagliflozin improved metabolic parameters and was associated with preservation of cortical microarchitecture. Similarly, in a T2DM mouse model, canagliflozin improved cortical bone geometry, though it did not fully restore bone strength or prevent trabecular bone loss, indicating that its skeletal benefits may extend beyond but are not solely mediated by glycemic improvement^28^. Another study evaluating dapagliflozin has also been shown to improve bone material properties independent of glycemic control^29^. These experimental findings may provide biological plausibility for the observed site‐specific association with hip fracture. However, they should not be interpreted as evidence of a broad protective effect of SGLT2i on skeletal outcomes.

Several limitations of this study should be considered. First, as an observational study, causality cannot be definitively established. Despite PSM and sensitivity analyses, residual confounding may remain. Second, key clinical variables such as glycated hemoglobin (HbA1c) and estimated glomerular filtration rate (eGFR) were unavailable in the claims database. To address this, we utilized validated surrogate indices, such as CCI and aDCSI, to approximate disease burden and diabetes severity. Nevertheless, prior studies have shown that patients initiating SGLT2i and DPP4i generally have comparable baseline HbA1c levels and achieve similar glycemic control after treatment^7^. Moreover, comparable fracture risks between the two drug classes have also been observed across different eGFR categories, suggesting that the absence of these laboratory measures is unlikely to substantially bias our results^30^. Third, follow‐up began 1 year after the index date to ensure adequate ascertainment of treatment exposure prior to outcome assessment. Consequently, patients who experienced a fracture, initiated anti‐osteoporotic medication, or died during this period were excluded. This design may have introduced selection bias by preferentially including individuals who remained event‐free during the first year after treatment initiation, as well as immortal time bias. However, this approach was intended to minimize the influence of pre‐existing skeletal fragility and fractures occurring before adequate treatment exposure had been established. The potential impact of differential exclusions between the SGLT2i and DPP4i groups could not be assessed and should therefore be considered when interpreting the study findings. Accordingly, our results should be interpreted as reflecting the comparative fracture risk among patients who survived and remained free of fracture and anti‐osteoporotic treatment during the first year after treatment initiation. In addition, incident and prevalent new users may differ in baseline clinical characteristics because of differences in treatment history and disease duration. However, these potential differences could not be fully characterized in the present study because baseline characteristics were not presented separately. Although subgroup analyses suggested some variation in treatment effects, formal tests for interaction were not statistically significant. Therefore, these findings should be interpreted cautiously and require confirmation in future studies. Fourth, the use of administrative claims data may introduce misclassification bias. Use of diagnostic, procedure, and prescription records likely improved case ascertainment. Fifth, while high‐risk individuals (e.g., those with prior fractures or anti‐osteoporotic medication use) were excluded to minimize confounding by pre‐existing skeletal fragility, this approach may limit generalizability to such high‐risk patients. These patients likely represent a minority of the T2DM population. Sixth, while prescription refill data were used to estimate medication adherence, they do not directly capture actual drug intake behavior, potentially leading to exposure misclassification. Although the analytical approach used in this study is commonly applied in PSM observational studies, recent methodological studies suggest that accounting for the matched structure may yield more appropriate variance estimates. In our study, however, the Fine‐Gray subdistribution hazard models did not explicitly account for the matched nature of the data when estimating variance. Consequently, the standard errors may have been underestimated, resulting in confidence intervals that are too narrow and P values that are smaller than expected. Therefore, our findings should be interpreted with appropriate caution. Multiple fracture outcomes and subgroup analyses were evaluated in this study. Although hip fracture was considered a clinically important outcome because it represents the most consequential osteoporotic fracture, the observed association was no longer statistically significant after a Benjamini‐Hochberg adjustment for multiple comparisons. Because the fracture outcomes are clinically related and likely correlated, this adjustment may have been conservative. Nevertheless, the possibility of a chance finding cannot be excluded, and the observed association should therefore be interpreted cautiously. Lastly, although this nationwide cohort provides robust real‐world evidence on the comparative skeletal safety of SGLT2i in Taiwanese patients with T2DM, the generalizability of our findings to other populations and healthcare settings warrants further investigation through multinational studies.

In conclusion, in this nationwide cohort that reflects routine clinical practice, SGLT2i therapy was not associated with an increased risk of overall or major osteoporotic fractures compared with DPP4i therapy. By including both incident new users and patients with prior DPP4i exposure, the prevalent new‐user design extends the evaluation beyond treatment‐naïve populations and better reflects real‐world treatment patterns. Although lower SHRs were observed in several prespecified subgroups, these findings should be considered exploratory because formal interaction tests were not statistically significant. Overall, the observed association with hip fracture warrants further investigation to determine whether it represents a true site‐specific association or a chance finding.

## DISCLOSURE

The authors declare no conflicts of interest related to this work.

Approval of the research protocol: The protocol for this research project has been approved by a suitably constituted Ethics Committee of National Yang Ming Chiao Tung University, and it conforms to the provisions of the Declaration of Helsinki (as revised in Fortaleza, Brazil, October 2013). Institutional Review Board of National Yang Ming Chiao Tung University, Approval No. NYCU113050AE.

Informed Consent: This study used de‐identified secondary data from the Taiwan National Health Insurance Research Database; therefore, the requirement for informed consent was waived by the Institutional Review Board.

Approval date of Registry and the Registration No. of the study/trial: N/A. This was a retrospective observational study using secondary administrative claims data and was not subject to prospective trial registration.

Animal Studies: N/A.

## Supporting information


**Table S1** Disease diagnostic codes and NHI payment codes for fracture outcomes.
**Table S2**. Disease diagnostic codes for comorbidities.
**Table S3**. ATC codes for medications.
**Table S4**. The incidence rates (per 1,000 PY) and hazard ratios of fracture outcomes.
**Table S5**. The subdistribution hazard ratios of fracture outcomes based on IPTW.
**Table S6**. The subdistribution hazard ratios of fracture outcomes based on as‐treated analysis.

## Data Availability

The data that support the findings of this study are available from Health and Welfare Data Science Center (HWDC), Taiwan. Restrictions apply to the availability of these data, which were used under license for this study. Data are available from the author(s) with the permission of Health and Welfare Data Science Center (HWDC), Taiwan.
