# Supplementary material for: SGLT2i use and fracture risk compared with DPP4i in patients with T2DM
Source: J Diabetes Investig. 2026 Sep 16:10.1111/jdi.70433. Online ahead of print. doi: 10.1111/jdi.70433 (PMC13579429; doi:10.1111/jdi.70433)
Supplement: Supplementary file 1 [file JDI-9999-0-s001.docx]

| Table S1. Disease diagnostic codes and NHI payment codes for fracture outcomes | | | |
| --- | --- | --- | --- |
| Fracture site | ICD-9-CM | ICD-10-CM | NHI payment code |
| Hip | 820.0, 820.2, 820.8, 821.0, 821.2 | S72001A, S72002A, S72009A, S72011A, S72012A, S72019A, S72021A, S72022A, S72023A, S72024A, S72025A, S72026A, S72031A, S72032A, S72033A, S72034A, S72035A, S72036A, S72041A, S72042A, S72043A, S72044A, S72045A, S72046A, S72051A, S72052A, S72059A, S72061A, S72062A, S72063A, S72064A, S72065A, S72066A, S72091A, S72092A, S72099A, S72101A, S72102A, S72109A, S72111A, S72112A, S72113A, S72114A, S72115A, S72116A, S72121A, S72122A, S72123A, S72124A, S72125A, S72126A, S72131A, S72132A, S72133A, S72134A, S72135A, S72136A, S72141A, S72142A, S72143A, S72144A, S72145A, S72146A, S7221XA, S7222XA, S7223XA, S7224XA, S7225XA, S7226XA, S72301A, S72302A, S72309A, S72321A, S72322A, S72323A, S72324A, S72325A, S72326A, S72331A, S72332A, S72333A, S72334A, S72335A, S72336A, S72341A, S72342A, S72343A, S72344A, S72345A, S72346A, S72351A, S72352A, S72353A, S72354A, S72355A, S72356A, S72361A, S72362A, S72363A, S72364A, S72365A, S72366A, S72391A, S72392A, S72399A, S72401A, S72402A, S72409A, S72411A, S72412A, S72413A, S72414A, S72415A, S72416A, S72421A, S72422A, S72423A, S72424A, S72425A, S72426A, S72431A, S72432A, S72433A, S72434A, S72435A, S72436A, S72441A, S72442A, S72443A, S72444A, S72445A, S72446A, S72451A, S72452A, S72453A, S72454A, S72455A, S72456A, S72461A, S72462A, S72463A, S72464A, S72465A, S72466A, S72471A, S72472A, S72479A, S72491A, S72492A, S72499A, S728X1A, S728X2A, S728X9A, S7290XA, S7291XA, S7292XA, S79001A, S79002A, S79009A, S79011A, S79012A, S79019A, S79091A, S79092A, S79099A, S79101A, S79102A, S79109A, S79111A, S79112A, S79119A, S79121A, S79122A, S79129A, S79131A, S79132A, S79139A, S79141A, S79142A, S79149A, S79191A, S79192A, S79199A | 64029B, 64030B, 64041C, 97836K, 97837A, 97838B, 64170B |
| Vertebral | 805.0, 805.2, 805.4, 805.6, 805.8, 806.0, 806.2, 806.4, 806.6, 806.8 | S12000A, S12001A, S1201XA, S1202XA, S12030A, S12031A, S12040A, S12041A, S12090A, S12091A, S12100A, S12101A, S12110A, S12111A, S12112A, S12120A, S12121A, S12130A, S12131A, S1214XA, S12150A, S12151A, S12190A, S12191A, S12200A, S12201A, S12230A, S12231A, S1224XA, S12250A, S12251A, S12290A, S12291A, S12300A, S12301A, S12330A, S12331A, S1234XA, S12350A, S12351A, S12390A, S12391A, S12400A, S12401A, S12430A, S12431A, S1244XA, S12450A, S12451A, S12490A, S12491A, S12500A, S12501A, S12530A, S12531A, S1254XA, S12550A, S12551A, S12590A, S12591A, S12600A, S12601A, S12630A, S12631A, S1264XA, S12650A, S12651A, S12690A, S12691A, S129XXA, S14101A, S14102A, S14103A, S14104A, S14105A, S14106A, S14107A, S14109A, S14111A, S14112A, S14113A, S14114A, S14115A, S14116A, S14117A, S14121A, S14122A, S14123A, S14124A, S14125A, S14126A, S14127A, S14131A, S14132A, S14133A, S14134A, S14135A, S14136A, S14137A, S14151A, S14152A, S14153A, S14154A, S14155A, S14156A, S14157A, S22000A, S22001A, S22002A, S22008A, S22009A, S22010A, S22011A, S22012A, S22018A, S22019A, S22020A, S22021A, S22022A, S22028A, S22029A, S22030A, S22031A, S22032A, S22038A, S22039A, S22040A, S22041A, S22042A, S22048A, S22049A, S22050A, S22051A, S22052A, S22058A, S22059A, S22060A, S22061A, S22062A, S22068A, S22069A, S22070A, S22071A, S22072A, S22078A, S22079A, S22080A, S22081A, S22082A, S22088A, S22089A, S24101A, S24102A, S24103A, S24104A, S24109A, S24111A, S24112A, S24113A, S24114A, S24131A, S24132A, S24133A, S24134A, S24151A, S24152A, S24153A, S24154A, S32000A, S32001A, S32002A, S32008A, S32009A, S32010A, S32011A, S32012A, S32018A, S32019A, S32020A, S32021A, S32022A, S32028A, S32029A, S32030A, S32031A, S32032A, S32038A, S32039A, S32040A, S32041A, S32042A, S32048A, S32049A, S32050A, S32051A, S32052A, S32058A, S32059A, S3210XA, S32110A, S32111A, S32112A, S32119A, S32120A, S32121A, S32122A, S32129A, S32130A, S32131A, S32132A, S32139A, S3214XA, S3215XA, S3216XA, S3217XA, S3219XA, S322XXA, S34101A, S34102A, S34103A, S34104A, S34105A, S34109A, S34111A, S34112A, S34113A, S34114A, S34115A, S34119A, S34121A, S34122A, S34123A, S34124A, S34125A, S34129A, S34131A, S34132A, S34139A, S343XXA | 33126B, 33127B, 64042C, 64160B |
| Radius | 813.0, 813.2, 813.4, 813.8 | S52001A, S52002A, S52009A, S52011A, S52012A, S52021A, S52022A, S52023A, S52024A, S52025A, S52026A, S52031A, S52032A, S52033A, S52034A, S52035A, S52036A, S52041A, S52042A, S52043A, S52044A, S52045A, S52046A, S52091A, S52092A, S52099A, S52101A, S52102A, S52109A, S52111A, S52112A, S52121A, S52122A, S52123A, S52124A, S52125A, S52126A, S52131A, S52132A, S52133A, S52134A, S52135A, S52136A, S52181A, S52182A, S52189A, S52201A, S52202A, S52209A, S52211A, S52212A, S52219A, S52221A, S52222A, S52223A, S52224A, S52225A, S52226A, S52231A, S52232A, S52233A, S52234A, S52235A, S52236A, S52241A, S52242A, S52243A, S52244A, S52245A, S52246A, S52251A, S52252A, S52253A, S52254A, S52255A, S52256A, S52261A, S52262A, S52263A, S52264A, S52265A, S52266A, S52271A, S52272A, S52279A, S52281A, S52282A, S52283A, S52291A, S52292A, S52299A, S52301A, S52302A, S52309A, S52311A, S52312A, S52319A, S52321A, S52322A, S52323A, S52324A, S52325A, S52326A, S52331A, S52332A, S52333A, S52334A, S52335A, S52336A, S52341A, S52342A, S52343A, S52344A, S52345A, S52346A, S52351A, S52352A, S52353A, S52354A, S52355A, S52356A, S52361A, S52362A, S52363A, S52364A, S52365A, S52366A, S52371A, S52372A, S52379A, S52381A, S52382A, S52389A, S52391A, S52392A, S52399A, S52501A, S52502A, S52509A, S52511A, S52512A, S52513A, S52514A, S52515A, S52516A, S52521A, S52522A, S52529A, S52531A, S52532A, S52539A, S52541A, S52542A, S52549A, S52551A, S52552A, S52559A, S52561A, S52562A, S52569A, S52571A, S52572A, S52579A, S52591A, S52592A, S52599A, S52601A, S52602A, S52609A, S52611A, S52612A, S52613A, S52614A, S52615A, S52616A, S52621A, S52622A, S52629A, S52691A, S52692A, S52699A, S5290XA, S5291XA, S5292XA, S59001A, S59002A, S59009A, S59011A, S59012A, S59019A, S59021A, S59022A, S59029A, S59031A, S59032A, S59039A, S59041A, S59042A, S59049A, S59091A, S59092A, S59099A, S59101A, S59102A, S59109A, S59111A, S59112A, S59119A, S59121A, S59122A, S59129A, S59131A, S59132A, S59139A, S59141A, S59142A, S59149A, S59191A, S59192A, S59199A, S59201A, S59202A, S59209A, S59211A, S59212A, S59219A, S59221A, S59222A, S59229A, S59231A, S59232A, S59239A, S59241A, S59242A, S59249A, S59291A, S59292A, S59299A | 64032B, 64044C, 64271B, 64271C, 97826K, 97827A, 97828B, 95014C, 95015C, 95002C, 95003C |
| Humerus | 812.0, 812.2, 812.4 | S42201A, S42202A, S42209A, S42211A, S42212A, S42213A, S42214A, S42215A, S42216A, S42221A, S42222A, S42223A, S42224A, S42225A, S42226A, S42231A, S42232A, S42239A, S42241A, S42242A, S42249A, S42251A, S42252A, S42253A, S42254A, S42255A, S42256A, S42261A, S42262A, S42263A, S42264A, S42265A, S42266A, S42271A, S42272A, S42279A, S42291A, S42292A, S42293A, S42294A, S42295A, S42296A, S42301A, S42302A, S42309A, S42311A, S42312A, S42319A, S42321A, S42322A, S42323A, S42324A, S42325A, S42326A, S42331A, S42332A, S42333A, S42334A, S42335A, S42336A, S42341A, S42342A, S42343A, S42344A, S42345A, S42346A, S42351A, S42352A, S42353A, S42354A, S42355A, S42356A, S42361A, S42362A, S42363A, S42364A, S42365A, S42366A, S42391A, S42392A, S42399A, S42401A, S42402A, S42409A, S42411A, S42412A, S42413A, S42414A, S42415A, S42416A, S42421A, S42422A, S42423A, S42424A, S42425A, S42426A, S42431A, S42432A, S42433A, S42434A, S42435A, S42436A, S42441A, S42442A, S42443A, S42444A, S42445A, S42446A, S42447A, S42448A, S42449A, S42451A, S42452A, S42453A, S42454A, S42455A, S42456A, S42461A, S42462A, S42463A, S42464A, S42465A, S42466A, S42471A, S42472A, S42473A, S42474A, S42475A, S42476A, S42481A, S42482A, S42489A, S42491A, S42492A, S42493A, S42494A, S42495A, S42496A, S4290XA, S4291XA, S4292XA, S49001A, S49002A, S49009A, S49011A, S49012A, S49019A, S49021A, S49022A, S49029A, S49031A, S49032A, S49039A, S49041A, S49042A, S49049A, S49091A, S49092A, S49099A, S49101A, S49102A, S49109A, S49111A, S49112A, S49119A, S49121A, S49122A, S49129A, S49131A, S49132A, S49139A, S49141A, S49142A, S49149A, S49191A, S49192A, S49199A | 64033B, 64043C , 97831K, 97832A, 97833B |
| Ankle | 824.0, 824.2, 824.4, 824.6, 824.8 | S82301A, S82302A, S82309A, S82391A, S82392A, S82399A, S8251XA, S8252XA, S8253XA, S8254XA, S8255XA, S8256XA, S8261XA, S8262XA, S8263XA, S8264XA, S8265XA, S8266XA, S82841A, S82842A, S82843A, S82844A, S82845A, S82846A, S82851A, S82852A, S82853A, S82854A, S82855A, S82856A, S82871A, S82872A, S82873A, S82874A, S82875A, S82876A, S82891A, S82892A, S82899A, S89101A, S89102A, S89109A, S89111A, S89112A, S89119A, S89121A, S89122A, S89129A, S89131A, S89132A, S89139A, S89141A, S89142A, S89149A, S89191A, S89192A, S89199A, S89301A, S89302A, S89309A, S89311A, S89312A, S89319A, S89321A, S89322A, S89329A, S89391A, S89392A, S89399A | 64046C, 64272C, 64273C, 95016C , 95017C, 95004C, 95005C, 95006C, 95007C |
| Clavicle | 810 | S42001A, S42001B, S42002A, S42002B, S42009A, S42009B, S42011A, S42011B, S42012A, S42012B, S42013A, S42013B, S42014A, S42014B, S42015A, S42015B, S42016A, S42016B, S42017A, S42017B, S42018A, S42018B, S42019A, S42019B, S42021A, S42021B, S42022A, S42022B, S42023A, S42023B, S42024A, S42024B, S42025A, S42025B, S42026A, S42026B, S42031A, S42031B, S42032A, S42032B, S42033A, S42033B, S42034A, S42034B, S42035A, S42035B, S42036A, S42036B | 64015C, 64016C |
| Pelvis | 808.0, 808.2, 808.4, 808.8 | S32301A, S32302A, S32309A, S32311A, S32312A, S32313A, S32314A, S32315A, S32316A, S32391A, S32392A, S32399A, S32401A, S32402A, S32409A, S32411A, S32412A, S32413A, S32414A, S32415A, S32416A, S32421A, S32422A, S32423A, S32424A, S32425A, S32426A, S32431A, S32432A, S32433A, S32434A, S32435A, S32436A, S32441A, S32442A, S32443A, S32444A, S32445A, S32446A, S32451A, S32452A, S32453A, S32454A, S32455A, S32456A, S32461A, S32462A, S32463A, S32464A, S32465A, S32466A, S32471A, S32472A, S32473A, S32474A, S32475A, S32476A, S32481A, S32482A, S32483A, S32484A, S32485A, S32486A, S32491A, S32492A, S32499A, S32501A, S32502A, S32509A, S32511A, S32512A, S32519A, S32591A, S32592A, S32599A, S32601A, S32602A, S32609A, S32611A, S32612A, S32613A, S32614A, S32615A, S32616A, S32691A, S32692A, S32699A, S32810A, S32811A, S3282XA, S3289XA, S329XXA | 64042C, 64161B |
| Tibia & fibula | 823.0, 823.2, 823.4, 823.8 | S82101A, S82102A, S82109A, S82111A, S82112A, S82113A, S82114A, S82115A, S82116A, S82121A, S82122A, S82123A, S82124A, S82125A, S82126A, S82131A, S82132A, S82133A, S82134A, S82135A, S82136A, S82141A, S82142A, S82143A, S82144A, S82145A, S82146A, S82151A, S82152A, S82153A, S82154A, S82155A, S82156A, S82161A, S82162A, S82169A, S82191A, S82192A, S82199A, S82201A, S82202A, S82209A, S82221A, S82222A, S82223A, S82224A, S82225A, S82226A, S82231A, S82232A, S82233A, S82234A, S82235A, S82236A, S82241A, S82242A, S82243A, S82244A, S82245A, S82246A, S82251A, S82252A, S82253A, S82254A, S82255A, S82256A, S82261A, S82262A, S82263A, S82264A, S82265A, S82266A, S82291A, S82292A, S82299A, S82311A, S82312A, S82319A, S82401A, S82402A, S82409A, S82421A, S82422A, S82423A, S82424A, S82425A, S82426A, S82431A, S82432A, S82433A, S82434A, S82435A, S82436A, S82441A, S82442A, S82443A, S82444A, S82445A, S82446A, S82451A, S82452A, S82453A, S82454A, S82455A, S82456A, S82461A, S82462A, S82463A, S82464A, S82465A, S82466A, S82491A, S82492A, S82499A, S82811A, S82812A, S82819A, S82821A, S82822A, S82829A, S82831A, S82832A, S82839A, S82861A, S82862A, S82863A, S82864A, S82865A, S82866A, S8291XA, S8292XA, S89001A, S89002A, S89009A, S89011A, S89012A, S89019A, S89021A, S89022A, S89029A, S89031A, S89032A, S89039A, S89041A, S89042A, S89049A, S89091A, S89092A, S89099A, S89201A, S89202A, S89209A, S89211A, S89212A, S89219A, S89221A, S89222A, S89229A, S89291A, S89292A, S89299A | 64031B, 64031C, 64043C, 97841K, 97842A, 97843B, 95016C, 95017C, 95004C, 95005C, 95006C, 95007C |
| Ribs | 807 | S128XXA, S2220XA, S2220XB, S2221XA, S2221XB, S2222XA, S2222XB, S2223XA, S2223XB, S2224XA, S2224XB, S2231XA, S2231XB, S2232XA, S2232XB, S2239XA, S2239XB, S2241XA, S2241XB, S2242XA, S2242XB, S2243XA, S2243XB, S2249XA, S2249XB, S225XXA, S225XXB | 64017C |
| Other | 818, 809, 811.0, 822.0, 819, 827, 828, 829 | S2220XA, S2249XA, S229XXA, S229XXB, S42101A, S42102A, S42109A, S42111A, S42112A, S42113A, S42114A, S42115A, S42116A, S42121A, S42122A, S42123A, S42124A, S42125A, S42126A, S42131A, S42132A, S42133A, S42134A, S42135A, S42136A, S42141A, S42142A, S42143A, S42144A, S42145A, S42146A, S42151A, S42152A, S42153A, S42154A, S42155A, S42156A, S42191A, S42192A, S42199A, S4290XA, S4291XA, S4292XA, S5290XA, S5291XA, S5292XA, S6290XA, S6290XB, S82001A, S82002A, S82009A, S82011A, S82012A, S82013A, S82014A, S82015A, S82016A, S82021A, S82022A, S82023A, S82024A, S82025A, S82026A, S82031A, S82032A, S82033A, S82034A, S82035A, S82036A, S82041A, S82042A, S82043A, S82044A, S82045A, S82046A, S82091A, S82092A, S82099A, S8290XA, S8290XB, S8290XC, S8291XA, S8291XB, S8291XC, S8292XA, S8292XB, S8292XC, T07, T148 |  |

Abbreviation: ICD-9-CM = International Classification of Diseases, Ninth Revision, Clinical Modification; ICD-10-CM = International Classification of Diseases, Tenth Revision, Clinical Modification; NHI=National Health Insurance

| Table S2. Disease diagnostic codes for comorbidities | | |
| --- | --- | --- |
| Comorbidities | ICD-9-CM | ICD-10-CM |
| Hypertension | 401, 402, 403, 404, 405, 437.2 | I10, I11, I12, I13, I15, I16, I67.4 |
| Hyperlipidemia | 272 | E78.5 |
| Ischemic heart disease | 410-414 | I20-I25 |
| Cardiac arrhythmia | 427 | I47.1 |
| Heart failure | 398.91, 402.01, 402.11, 402.91, 404.01, 404.03, 404.11, 404.13, 404.91, 404.93, 428.xx | I09.81, I11.0, I13.0, I13.2, I50.xx |
| Peripheral vascular diseases | 442.3, 440.21, 443.81, 443.9, 892.1, 040.0, 444.22, 785.4, 250.7x, 249.7, 707.1x | E08.51, E09.51, E10.51, E11.51, E13.51, E08.59, E09.59, E10.59, E11.59, E13.59, E08.621, E09.621, E10.621, E11.621, E13.621, I72.4, I73.89, I73.9, A48.0, I74.3, I96, E08.52, E09.52, E10.52, E11.52, E13.52, I70.21x, S91.3x, L97.x |
| Ischemic stroke | 433.x1, 434.x1, 435.9, 436, 437.1x, 437.9x | I63.X, I64.X, G45.9, I67.81, I67.82, I67.89, I67.9 |
| Chronic kidney disease | 580-587 | N18, N19 |
| Chronic lung disease | 490.x-505.x, 506.4 | I27.8, I27.9, J40.x-J47.x, J60.x-J67.x, J68.4, J70.1, J70.3 |
| Cancer | 140.xx-208.xx ,195.1-195.8 | C00.xx-C97.xx |
| Dementia | 046.1x, 290.1x, 290.2x, 290.3, 290.4x, 291.2, 292.82, 294.1x, 294.2x, 331.0, 331.1x, 331.2, 331.6, 331.82, 331.89 | A81.0x, F01.5x, F02.8x, F03.9x, F10.27, F10.97, F13.27, F13.97, F18.97, F19.17, F19.27, F19.97, G30.x, G31.0x, G31.1, G31.83, G31.85 |
| Hypovolemia | 276.5, 276.50, 276.52, 276.51 | E86.1, E86.9, E86.0, R57.1 |

Abbreviation: ICD-9-CM = International Classification of Diseases, Ninth Revision, Clinical Modification; ICD-10-CM = International Classification of Diseases, Tenth Revision, Clinical Modification

| Table S3. ATC codes for medications | |
| --- | --- |
| Medications | ATC code |
| DPP4i | A10BH, A10BD07, A10BD08, A10BD09, A10BD10, A10BD11, A10BD13 |
| SGLT2i | A10BK, A10BD15, A10BD20 |
| Metformin | A10BA02, A10BA03, A10BD02, A10BD03, A10BD05, A10BD07, A10BD08, A10BD10, A10BD11, A10BD13, A10BD14, A10BD15, A10BD20 |
| SU | A10BB01, A10BB02, A10BB03, A10BB04, A10BB05, A10BB07, A10BB08, A10BB09, A10BB12, A10BB31, A10BD02 |
| GLP1-RA | A10BJ |
| Meglitinides | A10BX02, A10BX03 |
| TZD | A10BG |
| α-glucosidase inhibitors | A10BF01, A10BF02 |
| Insulin | A10A |
| ACEI | C09A |
| ARB | C09C |
| α-blockers | C02CA, C02LE |
| β-blockers | C07 |
| CCB | C08, C09BB, C09DX01, C10BX03 |
| Loop diuretics | C03C |
| Thiazide | C03AA |
| Potassium-sparing agents | C03D, C03E |
| Antiplatelets | B01AC |
| Statins | C10AA |
| NSAIDs | N04B, N04, AA01-AA04, BA02, BA03, BB01, BC01-BC06, BD01, BD02, BX01, BX02, N05A, B01AC06, N02BA01, N02BA51, M01A, N02BE01, B01AC06, N02BA01, N02BA51, B01AA03, B01AA04, C01A-C01D, C07, C08, C09A-C09D, N05A |

Abbreviation: ACEI = angiotensin-converting enzyme inhibitor; ARB = angiotensin II receptor blocker; CCB = calcium channel blocker; DPP4i = dipeptidyl peptidase-4 Inhibitor; NSAID = non-steroidal anti-inflammatory drug; SGLT2i = sodium-glucose cotransporter-2 inhibitor; TZD = thiazolidinedione

| Table S4. The incidence rates (per 1000 PY) and hazard ratios of fracture outcomes | | | | | | | |
| --- | --- | --- | --- | --- | --- | --- | --- |
| Outcomes | **Exposure** | **PY** | **Incidence** | **(95% CI)** | **HR*** | **(95% CI)** | ***P value*** |
| All fractures | DPP4i | 743,539 | 9.65 | (9.43-9.88) | 1.00 | (Ref.) |  |
|  | SGLT2i | 372,448 | 9.72 | (9.41-10.0) | 1.01 | (0.96-1.05) | 0.786 |
| Hip, vertebral, and radius | DPP4i | 747,410 | 6.99 | (6.80-7.18) | 1.00 | (Ref.) |  |
|  | SGLT2i | 374,363 | 7.03 | (6.76-7.30) | 1.00 | (0.96-1.05) | 0.901 |
| Hip | DPP4i | 755,665 | 1.23 | (1.16-1.32) | 1.00 | (Ref.) |  |
|  | SGLT2i | 378,558 | 1.09 | (0.98-1.20) | 0.86 | (0.76-0.97) | 0.014 |
| Vertebral | DPP4i | 752,936 | 2.93 | (2.81-3.06) | 1.00 | (Ref.) |  |
|  | SGLT2i | 377,023 | 3.10 | (2.92-3.28) | 1.05 | (0.98-1.13) | 0.175 |
| Radius | DPP4i | 752,544 | 3.03 | (2.90-3.15) | 1.00 | (Ref.) |  |
|  | SGLT2i | 376,929 | 3.01 | (2.84-3.19) | 1.00 | (0.93-1.07) | 0.970 |

*HR was estimated based on conditional Cox regression analysis

Abbreviation: CI = confidence interval; DPP4i = dipeptidyl peptidase-4 inhibitor; HR = hazard ratio; PY = person-year; SGLT2i = sodium-glucose cotransporter-2 inhibitor

| Table S5. The subdistribution hazard ratios of fracture outcomes based on IPTW | | | | | |
| --- | --- | --- | --- | --- | --- |
| Outcomes | **Exposure** | **No. of Event** | **SHR*** | **(95% CI)** | ***P value*** |
| All fractures | DPP4i | 22,019 | 1.00 | (Ref.) |  |
|  | SGLT2i | 21,052 | 0.98 | (0.94-1.01) | 0.161 |
| Hip, vertebral, and radius | DPP4i | 16,534 | 1.00 | (Ref.) |  |
|  | SGLT2i | 15,666 | 0.97 | (0.93-1.01) | 0.106 |
| Hip | DPP4i | 3,466 | 1.00 | (Ref.) |  |
|  | SGLT2i | 2,623 | 0.76 | (0.68-0.86) | <0.001 |
| Vertebral | DPP4i | 7,115 | 1.00 | (Ref.) |  |
|  | SGLT2i | 7,013 | 1.00 | (0.94-1.07) | 0.927 |
| Radius | DPP4i | 6,645 | 1.00 | (Ref.) |  |
|  | SGLT2i | 6,575 | 1.01 | (0.95-1.08) | 0.673 |

*SHR was estimated based on conditional competing risk model analysis

Abbreviation: CI = confidence interval; DPP4i = dipeptidyl peptidase-4 inhibitor; IPTW = inverse probability of treatment weighting; SHR = subdistribution hazard ratio; SGLT2i = sodium-glucose cotransporter-2 inhibitor

| Table S6. The subdistribution hazard ratios of fracture outcomes based on as-treated analysis | | | | | |
| --- | --- | --- | --- | --- | --- |
| Outcomes | **Exposure** | **No. of Event** | **SHR*** | **(95% CI)** | ***P value*** |
| All fractures | DPP4i | 6,222 | 1.00 | (Ref.) |  |
|  | SGLT2i | 2,012 | 0.99 | (0.95-1.04) | 0.799 |
| Hip, vertebral, and radius | DPP4i | 4,542 | 1.00 | (Ref.) |  |
|  | SGLT2i | 1,424 | 0.98 | (0.93-1.03) | 0.370 |
| Hip | DPP4i | 818 | 1.00 | (Ref.) |  |
|  | SGLT2i | 191 | 0.82 | (0.71-0.95) | 0.007 |
| Vertebral | DPP4i | 1,912 | 1.00 | (Ref.) |  |
|  | SGLT2i | 611 | 1.02 | (0.94-1.11) | 0.650 |
| Radius | DPP4i | 1,975 | 1.00 | (Ref.) |  |
|  | SGLT2i | 664 | 0.99 | (0.92-1.07) | 0.796 |

*SHR was estimated based on conditional competing risk model analysis

Abbreviation: CI = confidence interval; DPP4i = dipeptidyl peptidase-4 inhibitor; SHR = subdistribution hazard ratio; SGLT2i = sodium-glucose cotransporter-2 inhibitor
